# Selective preparation of zero- and one-dimensional gold nanostructures in a TiO_2 _nanocrystal-containing photoactive mesoporous template

**DOI:** 10.1186/1556-276X-7-27

**Published:** 2012-01-05

**Authors:** Go Kawamura, Teruhisa Okuno, Hiroyuki Muto, Atsunori Matsuda

**Affiliations:** 1 Department of Electrical and Electronic Information Engineering, Toyohashi University of Technology, 1-1 Hibarigaoka, Tempaku-cho, Toyohashi, Aichi, 441-8580, Japan; 2 Department of Environmental and Life Sciences, Toyohashi University of Technology, 1-1 Hibarigaoka, Tempaku-cho, Toyohashi, Aichi, 441-8580, Japan

**Keywords:** mesoporous, titania, template, gold, nanostructures, shape control, photocatalysis, surface plasmon resonance

## Abstract

Nanocrystallized SiO_2_-TiO_2 _with tubular mesopores was prepared via the sol-gel technique. Gold was deposited in the tubular mesopores of the nanocrystallized SiO_2_-TiO_2_. The shape of the gold was varied from one-dimensional [1-D] to zero-dimensional [0-D] nanostructures by an increase in TiO_2 _content and ultraviolet [UV] irradiation during gold deposition. 1-D gold nanostructures [GNSs] were mainly obtained in the mesopores when a small amount of TiO_2_-containing mesoporous SiO_2_-TiO_2 _was used as a template, whereas the use of a template containing a large amount of TiO_2 _led to the formation of shorter 1-D or 0-D GNSs. UV irradiation also resulted in the formation of 0-D GNSs.

**PACS: **06.60.Jn (sample preparation); 81.07.Gf (nanowires); 81.16.Be (chemical synthesis methods).

## Introduction

Gold nanostructures [GNSs] have been attracting much attention because of the high chemical stability coincident with their unique optoelectronic properties, which are dependent on the morphology of the GNSs [1-4]. Surface plasmon resonance [SPR] is one of the most interesting properties of one-dimensional [1-D] GNSs [2-5]. The wavelength of SPR is affected by the length, diameter, and aspect ratio of the 1-D GNSs [6,7]. Aligned GNSs perform polarization of light [8-10]. Such multifunctionality of the 1-D GNSs opens up new application fields such as wavelength-sensitive nonlinear optical devices and polarization filters [8,9,11]. Several methods for synthesizing GNSs including 1-D GNSs have been reported. These methods include photochemical and electrochemical deposition [12,13] and seeding growth methods [14,15]. In these methods, however, the GNSs are suspended in a solvent. Therefore, the GNSs are required to be immobilized in a designed fashion in/on a solid matrix for various kinds of practical applications. The immobilization process for GNSs still requires further development [3,10,16].

On the other hand, the use of hard templates such as anodic alumina and mesoporous silica for the synthesis of 1-D GNSs makes the complicated immobilization processes redundant, and several related studies have been reported [17,18]. Those methods using hard templates are also advantageous to control the diameter and dispersion state of 1-D GNSs because they depend on the pore structure. However, methods that control the morphology of the 1-D GNSs have several problems. For example, the elongation of the 1-D GNSs requires more gold to be deposited in the template. This obstructs, for example, the investigation of the shape-dependent properties of the GNSs. Therefore, a novel method to control the morphology of the 1-D GNSs in hard templates without changing the gold amount is eagerly demanded.

In this work, nanocrystallized SiO_2_-TiO_2 _with tubular mesopores was prepared and used as an active template. 0-D and 1-D GNSs were deposited in the tubular mesopores. The shape of the GNSs was observed, and the SPR characteristics were measured. It is known that TiO_2 _nanocrystals generate electrons through heating and ultraviolet [UV] irradiation. In this study, the generated electrons were found to transfer to the Au^3+ ^ions. As such, the deposition rate of the GNSs can be controlled by controlling the amount of electrons generated. As a result, 0-D and 1-D GNSs are selectively deposited.

## Experimental methods

### Materials

Pluronic P123 ((EO)_20_(PO)_70_(EO)_20_, poly(ethylene oxide), and poly(propylene oxide)) was purchased from Sigma-Aldrich (St. Louis, MO, USA). Tetraethoxysilane [TEOS] and 3-aminopropyltriethoxysilane [APTES] were obtained from Shin-Etsu Chemical Co., Ltd. (Tokyo, Japan). Titanium tetra-*n*-butoxide [TTB] and HAuCl_4 _were acquired from Wako Pure Chemical Industries, Ltd. (Osaka, Japan) and Kishida (Osaka, Japan), respectively.

### Synthesis of mesoporous template

The preparation procedure of 20Ti is described as a typical example. A mixture of P123 (1.74 g), NaCl (2.92 g), and 1 mM HCl (100 mM) was added to TEOS (4.18 g) and stirred at 35°C for 24 h. TTB (1.70 g) was then added to the solution and stirred further for 6 h. For the preparation of (100-*x*)SiO_2_·*x*TiO_2_, only the ratio of TEOS to TTB was varied. The stirred solution was transferred into an autoclave vessel and kept at 100°C for 4 h. The precipitated powder was collected by suction filtration, then washed with ion-exchanged water [IEW] and ethanol, and dried in an ambient environment. The obtained powder was calcined at 550°C for 5 h to remove the surfactant from the mesopore.

### Loading of Au

The obtained powder was immersed in the 1 wt.% APTES solution (in ethanol) and stirred at 25°C for 3 h. The powder was then filtered with suction, washed with ethanol, and dried at 60°C in air. The amino-functionalized powder was mixed into a 1-mM HAuCl_4 _aqueous solution and stirred at 25°C for 2 h. After the suction filtration, the product was washed with IEW and dried in an ambient environment. The product was then calcined at 350°C for 3 h (at a heating rate of 1°C/min) with or without UV irradiation (USHIO SP-9, 230-440 nm, 2.5 mW/cm^2 ^at 365 nm).

### Characterization

X-ray diffraction [XRD] measurements were performed using a Rigaku RINT 2000 diffractometer (Rigaku Corporation, Tokyo, Japan) with CuKα radiation (*λ *= 1.5406 Å). Transmission electron microscopy [TEM] images and energy dispersive spectroscopy [EDS] were taken using a Hitachi H-800 transmission electron microscope (High-Technologies Corporation, Chiyoda, Tokyo, Japan) and a JEOL JEM-2100F (JEOL, Ltd., Akishima, Tokyo, Japan) transmission electron microscope operating at 200 kV. UV/visible-near infrared diffuse reflectance [Vis-NIR DR] spectra were measured using a JASCO V-670 UV-Vis-NIR spectroscope (JASCO Corporation, Tokyo, Japan).

## Results and discussion

**Figure 1:**
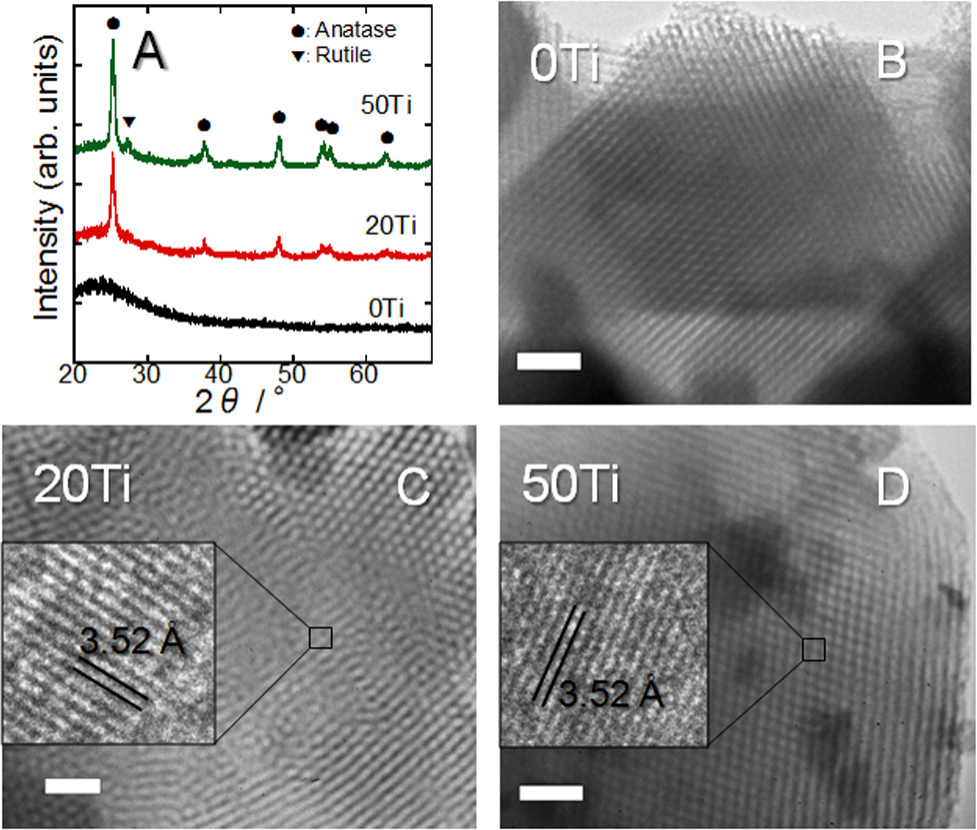
**XRD patterns and TEM images of 0Ti, 20Ti, and 50Ti**. (**A**) XRD patterns of 0Ti, 20Ti, and 50Ti. (**B**-**D**) The corresponding TEM images of the 0Ti, 20Ti, and 50Ti (scale bars, 50 nm). The insets in C and D are HR TEM images of the squared regions.

In the XRD pattern of the mesoporous 100SiO_2 _template [0Ti], amorphous SiO_2 _was observed as a halo at ca. 23°. On the other hand, the 80SiO_2_·20TiO_2 _template [20Ti] showed several peaks consistent with both anatase and rutile TiO_2_, as well as amorphous SiO_2 _(Figure 1A). The peaks of the TiO_2 _crystals appeared stronger in the pattern of the 50SiO_2_·50TiO_2 _template [50Ti] in comparison with those of 20Ti. A TEM observation of these templates revealed that all of them possessed a 2-D hexagonal mesoporous structure with the same caliber of ca. 7 nm (Figures 1B, C, D). The high-resolution TEM images of 20Ti and 50Ti showed ca. 4-nm crystals with a fringe spacing of 3.52 Å, which were well dispersed in the samples (insets of Figures 1C, D). The fringe spacing is consistent with the *d *value of {011} planes of anatase TiO_2_. This proves that the templates consist of pure amorphous SiO_2_, or SiO_2 _and well-dispersed TiO_2 _nanocrystals, forming a 2-D hexagonal mesoporous structure.

**Figure 2:**
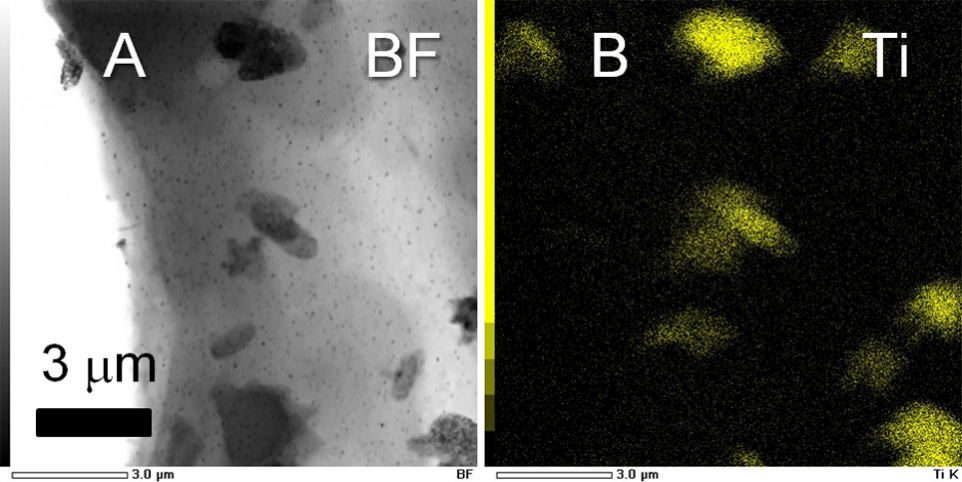
**TEM image and Ti elemental mapping of 20 Ti**. (**A**) Bright field TEM image and (**B**) Ti elemental mapping of 20Ti treated in water at 100°C for 24 h.

Particles without mesopores were rarely observed in the TEM images of 20Ti and 50Ti prepared by aging in water at 100°C for 4 h. On the other hand, 20Ti (and 50Ti) aged for 24 h in water at 100°C showed the formation of large nonporous particles outside the mesoporous structure (Figure 2A). The components of the nonporous particles and mesoporous region were solely attributed to TiO_2 _and SiO_2_, respectively (Figure 2B). Thus, the TiO_2 _crystal formation in our samples should be based on the dissolution and deposition of the TiO_2 _component by aging in hot water, where the TiO_2 _component in the SiO_2_-TiO_2 _gel system is dissolved into water and then reprecipitated as crystalline TiO_2 _[19,20]. The short aging time in hot water resulted in the suppression of large TiO_2 _particle formation and the sufficient deposition of TiO_2 _nanocrystals with a highly dispersed state on/in the mesoporous matrix. In this work, therefore, the aging time of 4 h was employed to prepare the templates, which possess almost the same pore size and structure regardless of the TiO_2 _content. The molar ratio of SiO_2 _to TiO_2 _in the mesoporous region was checked by EDS, and it was found to be comparable to the nominal molar ratio. By using the templates, the effect of the TiO_2 _nanocrystals on the shape of the 1-D GNSs can be investigated due to the sufficient formation of interfaces between the deposited Au and TiO_2 _nanocrystals.

**Figure 3:**
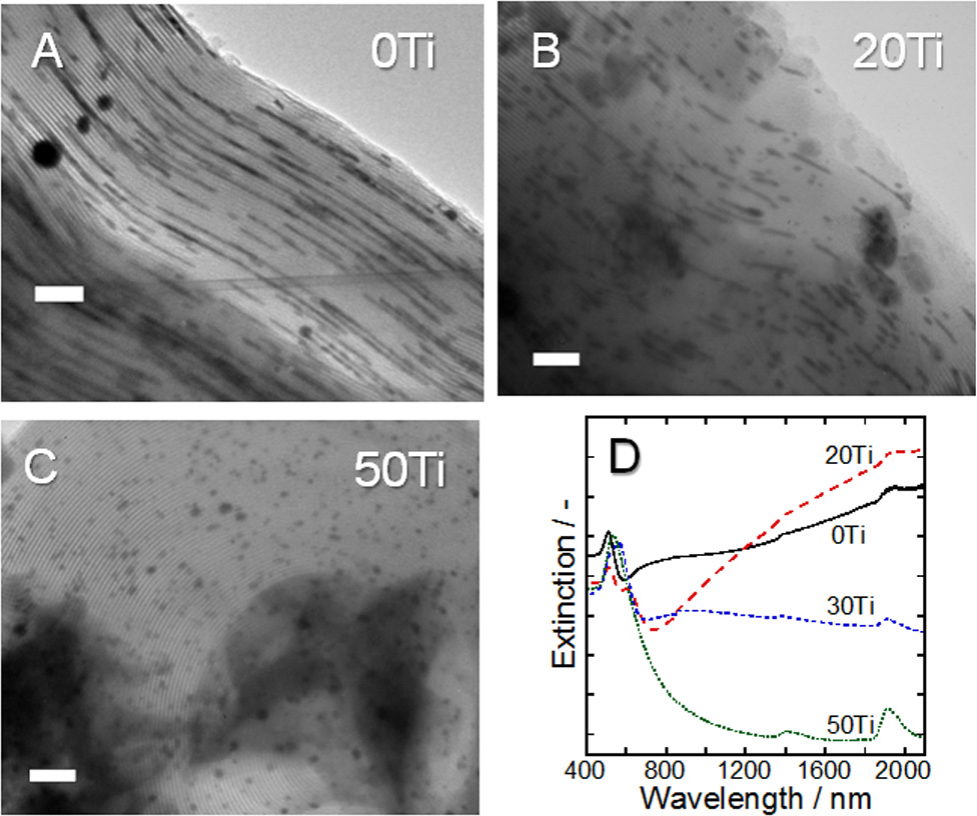
**TEM images and DR spectra after Au deposition**. TEM images of (**A**) 0Ti, (**B**) 20Ti, and (**C**) 50Ti after Au deposition (scale bars, 100 nm). (**D**) The corresponding DR spectra.

Long 1-D GNSs were formed in 0Ti after Au loading (Figure 3A). At the beginning of the Au loading, the Au^3+ ^ions adsorb to the amino groups on the wall of the mesopores. Heat treatment of the resultant powder causes decomposition of the amino group-containing organic matter. The Au^3+ ^ions are released and partly reduced to Au^+ ^ions and Au atoms by electrons provided from the decomposed organic matter. The Au atoms agglomerate and form Au nanoclusters, and then the Au ions released from the amino groups are reduced on the Au nanoclusters by autocatalysis of Au [21,22], causing the Au metal to grow. Since the growth of Au occurs in the tubular mesopores, the final shape of the Au should be 1-D or 0-D GNSs. A morphology change of the GNSs was then investigated when the content of TiO_2 _in the template was varied. The length of the 1-D GNSs formed in 20Ti (Figure 3B) was shorter than that deposited in 0Ti, whereas 0-D GNSs were predominantly obtained in 50Ti (Figure 3C). These results indicate that an increase in TiO_2 _content leads to a shortening of the 1-D GNSs. This is presumably because thermoexcited conduction electrons are generated from TiO_2_, and these generated electrons transfer to the Au ions to accelerate their reduction [23]. TiO_2 _heated at 350°C generates approximately 8.8 × 10^13 ^times as many thermoexcited electrons as TiO_2 _does at room temperature [24]. Therefore, the amount of electrons supplied to the Au ions increases as the TiO_2 _content increases. As a result, Au metal is rapidly deposited prior to its migration for the formation of long 1-D GNSs. Therefore, 0-D GNSs were predominantly formed in the tubular mesopores by using templates containing more than 50 mol% TiO_2_.

1-D GNSs deposited in 0Ti showed two extinction peaks in the diffuse reflectance [DR] spectrum: a sharp extinction peak at 500 nm and a broad extinction peak spreading over the whole region of the NIR region (Figure 3D). The shorter- and longer-wavelength extinctions are attributed to the transverse mode of SPR and the light scattering by fairly long 1-D GNSs [25], respectively. The length of the 1-D GNSs was shortened when 20Ti was used. An extinction peak appeared at around 600 nm, and the extinction intensity at wavelengths longer than 1,200 nm increased. This is presumably due to the shortening of the 1-D GNSs, which leads to a decrease in the light scattering intensity of the long 1-D GNSs (appearing over the whole NIR region) and an increase in the longitudinal SPR [LSPR] mode caused by the short 1-D GNSs (appearing at the NIR region toward the shorter wavelength side, e.g. 600 nm and approximately 2,000 nm in this case). With 30Ti, the LSPR peaks blue-shifted and appeared at 580 and approximately 900 nm. When 50Ti was used, only 0-D GNSs were deposited accompanied by a 520-nm peak, which is attributed to the SPR of the 0-D GNSs. By the use of a mesoporous SiO_2 _template containing less than 30 mol% TiO_2_, 1-D GNSs exhibiting LSPR, which is excited by NIR light, are deposited regardless of the presence of TiO_2 _nanocrystallites in the template.

**Figure 4:**
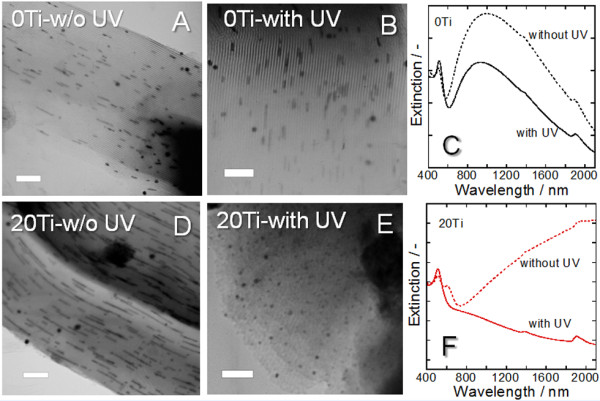
**TEM images of 0Ti and 20Ti with their corresponding DR spectra after Au deposition**. TEM images of 0Ti after thermal Au deposition were carried out (**A**) Without or (**B**) with simultaneous UV irradiation. (**C**) The corresponding DR spectra of A and B. TEM images of 20Ti after thermal Au deposition was carried out (**D**) without or (**E**) with simultaneous UV irradiation. (**F**) The corresponding DR spectra of D and E. (scale bars, 100 nm).

Since TiO_2 _is known as a photocatalyst that generates electrons and holes by UV irradiation, the effects of UV irradiation during Au loading on the shape of the GNSs were investigated. As for the TiO_2 _crystalline phases, anatase TiO_2 _was widely recognized as the most suitable phase for photocatalysis [26,27]; but recent reports suggest that mixed rather than single phases can be even more active [28,29]. Also, since the TiO_2 _nanocrystals in our samples possess diameters of a few nanometers, which are almost the optimum size for photocatalysis [26], UV irradiation of the templates should generate charges and influence the shape of the GNSs. In the case where 0Ti was used, 1-D GNSs with a length of 10 to 100 nm were deposited regardless of the UV irradiation (Figures 4A, B). It is worth mentioning that the 1-D GNSs in 0Ti were fabricated with a short length by shortening the calcination time in order to discuss the shift of the resonant wavelength in the measurable NIR region. In the DR spectra, the LSPR wavelengths of the samples prepared with and without UV irradiation were 930 and 990 nm, respectively. The wavelengths were slightly shifted, and the LSPR extinction intensity decreased slightly upon UV irradiation (Figure 4C). The results reveal that UV irradiation has only a minor effect on the shape of the deposited 1-D GNSs when 0Ti is used. On the other hand, although 1-D GNSs with a length of 10 to 400 nm were obtained in 20Ti without UV irradiation (Figure 4D), 0-D GNSs were predominantly deposited when UV irradiation was carried out (Figure 4E). The extinction peaks in the NIR region almost disappeared when the sample was exposed to UV irradiation (Figure 4F). These results clearly show that UV irradiation during Au loading in 20Ti influences the shape of the GNSs. Furthermore, since the GNSs are insensitive toward UV light, it is evident that the photocatalytic activity of TiO_2 _affects the shape of the GNSs in the template.

**Figure 5:**
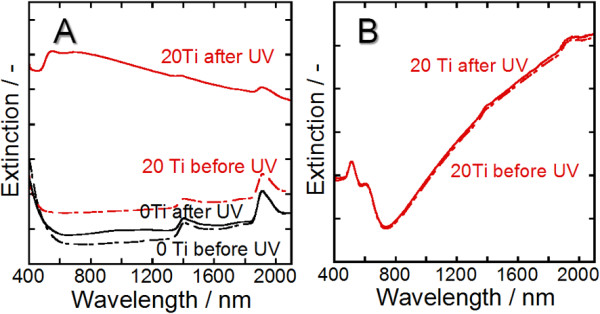
**DR spectra of Au^3+ ^ion-adsorbed 20Ti and 0Ti and of 1-D GNSs**. (**A**) DR spectra of Au^3+ ^ion-adsorbed 20Ti and 0Ti before and after UV irradiation (no heat treatment). (**B**) DR spectra of the 1-D GNSs thermally pre-deposited in 20Ti before and after UV irradiation.

Two mechanisms were considered for the preferential formation of 0-D GNSs by UV irradiation: acceleration of the Au-ion reduction rate by the generated electrons or oxidation of deposited Au metal by the generated holes. In the case where UV irradiation at room temperature was carried out on the Au ion-adsorbed 20Ti, an increase in extinction intensity was observed from the Vis to the NIR region (Figure 5A). The increased extinction was attributed to the formation of 1-D GNSs of various lengths because of the wide wavelength region of SPR. On the other hand, UV irradiation of 0Ti resulted in a slight increase in extinction intensity over a similarly wide wavelength region (Figure 5A). This is probably due to the marginal deposition of GNSs by UV irradiation, which partly decomposes the organic matter adsorbed on the mesoporous wall, and thus, a small number of electrons are generated that reduce Au ions. This would explain the spectral change in Figure 4C, where the thermal and photo decomposition of the organic matter occurs simultaneously at the beginning of the Au loading process; thus, the Au reduction rate is slightly increased. Furthermore, since the variation of the extinction intensity in 20Ti is much larger than that in 0Ti, it is clear that UV irradiation accelerates the reduction of Au ions as a result of the electrons generated from TiO_2_. On the other hand, UV irradiation after the thermal deposition of 1-D GNSs in 20Ti led to little change in the DR spectra (Figure 5B). This indicates that the holes, which are expected to oxidize the deposited 1-D GNSs to Au ions, have negligible effect on the shape change in the GNSs. Thus, it is concluded that the photocatalysis of TiO_2 _causes the reduction of the gold ions rather than oxidation of the Au metal. The generated holes may be consumed to decompose organic matter adsorbed on the wall of the template. In addition, UV irradiation of the 1-D GNSs deposited in 0Ti also resulted in no change in the DR spectra.

The results obtained suggest the probable mechanism of Au deposition by the simultaneous heat treatment and UV irradiation, where the predominant formation of 0-D GNSs was observed (Figure 4E). The heat treatment causes the decomposition of organic matter adsorbed on the wall of the template. This results in the partial reduction of Au^3+ ^ions, followed by the formation of scattered Au nanoclusters. The partially reduced Au ions are released from their electrostatic adsorption to the amino groups and associated with the oxygen atoms on the wall surface of the matrix, enabling mobility of the Au ions [30]. The Au ions, therefore, can reach the neighboring Au nanoclusters and are reduced on the surface of the nanoclusters by autocatalysis of Au [21,22], resulting in the formation of 1-D GNSs because the growth of Au occurs in the tubular mesopores. Thermally excited electrons of TiO_2 _accelerate the reduction rate of the Au ions; thus, a large content of TiO_2 _in the template leads to the preferential formation of 0-D GNSs. Furthermore, UV irradiation also accelerates the reduction rate of the Au ions. Therefore, the combination of heat treatment and UV irradiation leads to a fast rate of Au deposition. The time taken for the movement of the Au ions is shortened, and the formation of 0-D GNSs instead of 1-D GNSs becomes dominant. By optimizing the heating and UV irradiation condition of our method, 1-D and 0-D GNSs are selectively deposited regardless of the composition of the template, where the amount of deposited Au atoms is constant but the shape of the GNSs is different.

## Conclusion

We have demonstrated the preparation of TiO_2 _nanocrystal-containing mesoporous templates and deposited 1-D or 0-D GNSs in the as-formed tubular mesopores. Since the provision of thermally generated electrons from TiO_2 _increased when a template contained a large amount of TiO_2_, Au ions were rapidly reduced and deposited as shorter 1-D or 0-D GNSs. Similarly, UV irradiation during Au deposition in the TiO_2_-containing template produced electrons photocatalytically and accelerated the Au deposition rate, leading to the dominant formation of 0-D GNSs.

## Abbreviations

APTES: 3-aminopropyltriethoxysilane; DR: diffuse reflectance; EDS: energy dispersive spectroscopy; GNSs: gold nanostructures; HR: high-resolution; IEW: ion-exchanged water; LSPR: longitudinal surface plasmon resonance; NIR: near infrared; 1-D: one-dimensional; SPR: surface plasmon resonance; TEOS: tetraethoxysilane; TEM: transmission electron microscopy; TTB: titanium tetra-*n*-butoxide; UV: ultraviolet; Vis: visible; XRD: X-ray diffraction.

## Competing interests

The authors declare that they have no competing interests.

## Authors' contributions

GK, TO, and AM designed the study. TO performed the experiments with help from GK and AM. GK and AM contributed in drafting the manuscript. All authors edited and approved the manuscript.
